# Acquiring Authentic Data in Unattended Wireless Sensor Networks

**DOI:** 10.3390/s100402770

**Published:** 2010-03-26

**Authors:** Chia-Mu Yu, Chi-Yuan Chen, Chun-Shien Lu, Sy-Yen Kuo, Han-Chieh Chao

**Affiliations:** 1 Institute of Information Science, Academia Sinica, Taipei, Taiwan; E-Mail: r91045@csie.ntu.edu.tw (C.-M.Y.); lcs@iis.sinica.edu.tw (C.-S.L.); 2 Department of Electrical Engineering, National Taiwan University, Taipei, Taiwan; E-Mail: sykuo@cc.ee.ntu.edu.tw; 3 Department of Electrical Engineering, National Dong Hwa University, Hualian, Taiwan; E-Mail: chiyuan.chen@gmail.com (C.-Y.C.); 4 Department of Electronic Engineering and Institute of Computer Science & Information Engineering, National Ilan University, I-Lan, Taiwan; E-Mail: hcc@niu.edu.tw (H.-C.C.)

**Keywords:** unattended wireless sensor network, UWSN, authentication

## Abstract

An Unattended Wireless Sensor Network (UWSN) can be used in many applications to collect valuable data. Nevertheless, due to the unattended nature, the sensors could be compromised and the sensor readings would be maliciously altered so that the sink accepts the falsified sensor readings. Unfortunately, few attentions have been given to this authentication problem. Moreover, existing methods suffer from different kinds of DoS attacks such as Path-Based DoS (PDoS) and False Endorsement-based DoS (FEDoS) attacks. In this paper, a scheme, called AAD, is proposed to Acquire Authentic Data in UWSNs. We exploit the collaboration among sensors to address the authentication problem. With the proper design of the collaboration mechanism, AAD has superior resilience against sensor compromises, PDoS attack, and FEDoS attack. In addition, compared with prior works, AAD also has relatively low energy consumption. In particular, according to our simulation, in a network with 1,000 sensors, the energy consumed by AAD is lower than 30% of that consumed by the existing method, ExCo. The analysis and simulation are also conducted to demonstrate the superiority of the proposed AAD scheme over the existing methods.

## 1. Introduction

The use of wireless sensor networks (WSNs) on data gathering applications has been popularized in recent years. Since WSNs could be deployed in the hostile environments, one of the fundamental issues is how to secure the collected data. Unfortunately, with the consideration of the sensors’ scarce resources, the security issue becomes very challenging because conventional computationally-intensive cryptographic primitives cannot be utilized.

**Figure 1.**
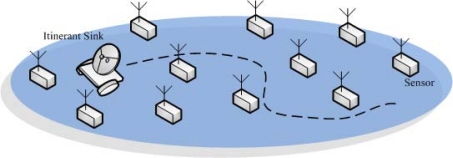
In a UWSN, the itinerant sink roams around the sensing region and collects the data sensed by sensors.

WSNs considered in the literature are usually assumed to have a constant connection between sensors and a trusted data collection unit, e.g., the sink. From the security point of view, in such a scenario, sensors can collect and then report the sensitive data to the sink at will. With the cryptographic primitives such as encryption and authentication, the confidentiality and authenticity of the transmitted sensed data can be guaranteed. More importantly, this avoid storing a large amount of data in sensors that are easily to be compromised. Even more, with the aid of the always-present sink, the network can defend against the attacks such as sensor compromises more efficiently and effectively. Nevertheless, due to the application restrictions, the above scenario is not always the case. In real world applications, there could be the cases where after the sensor deployment, the sensed data should be temporarily stored in the sensors because the sink is away from the network in most of the sensor network lifetime. Only at the end of each *collection interval*, will the itinerant sink roams around the sensing region and collects the data sensed by sensors. In contrast to the usual sensor networks, to emphasize the unattended WSN feature, this type of WSN consisting of sensors and an itinerant sink that periodically collects sensed data is termed as *unattended wireless sensor networks* (UWSNs). In fact, UWSNs have been used in practical WSN applications [1,2]. In particular, the unattended sensor networks in [1] operate in an unmanned manner. For example, the nuclear emission sensor network could be deployed to monitor potential nuclear activity. In addition, another example is to deploy unattended sensors to detect underground sound and vibration, in order to be aware of troop movements, border crossings, and enemy’s aircrafts as soon as possible. Trident systems [2] deploy the unattended ground sensors for providing reliable communication links. It is often used for transmitting timely message back to command and control centers. These sensors can be used in battlefield applications including perimeter defense, border patrol and surveillance, target acquisition, and situation awareness. The conceptional illustration is shown in Figure 1.

Due to its inability to offload the sensed data in a real-time manner, sensors should keep the data sensed in the local memory within the collection interval between successive sink visits, incentivizing various attacks. The adversary may have different goals; it may be interested in learning the data sensed by a specific set of sensors, or want to prevent certain data from reaching the data sink. In this paper, we consider the adversary whose goal is to alter the sensors’ data so that the falsified data can mislead the sink. In spite of the paramount importance of this authentication problem, only few solutions [3,4] are proposed. Specifically, in [3], a novel authentication function was proposed to deal with the authentication problem in a storage-efficient manner. Nevertheless, it is effective only against the *reactive adversary* (described in Section 3.2) that is relatively weak and is easily to be overcame. In [4], two collaborative authentication schemes, CoMAC and ExCo, were proposed to defend against the stronger adversary, *proactive adversary* (described in Section 3.2).

Unfortunately, the simple collaboration among the sensors in CoMAC and ExCo incurs more attacks such as Path-Based DoS (PDoS) [5] and False-Endorsement DoS (FEDoS) [6] attacks (described in Section 4.1). In addition, the resilience of ExCo against sensor compromises is not as strong as [4] claims (described in Section 4.1). Furthermore, due to the lack of the proper use of sensors’ position information, CoMAC and ExCo have relatively high energy consumption especially in a large scale network. The above reasons motivate us to develop a secure and efficient collaborative authentication scheme in UWSNs.

### 1.1. Contribution

We identify the security flaws of the schemes in [4]. Aiming at solving the identified problems, a scheme, called AAD, is proposed to Acquire Authentic Data in UWSNs. AAD possesses three characteristics. (1) Due to the proper use of sensors’ position information, it is communication-efficient. (2) In addition to acquiring authentic data, AAD is also resilient against both Path-Based DoS (PDoS) [5] and False-Endorsement DoS (FEDoS) [6] attacks. (3) The resilience of AAD against sensor compromises is superior to that in prior works [4]. From analytical and simulation results, the robustness of AAD is demonstrated to be superior to those of CoMAC and ExCo.

## 2. Related Work

Due to the use of Bloom filter in our proposed AAD scheme, its brief introduction is given in Section 2.1 Then, some related works performed on UWSNs are briefly described in Section 2.2

### 2.1. Bloom Filter

As a kind of probabilistic data structure, a Bloom filter consists of an array of *n* bits. Together with *k* independently and randomly selected hash functions, *h*_1_, ⋯, *h_k_*, with range [0, *n* − 1], it is used to represent a set of elements with the support of membership query. Assume that a Bloom filter *B* is used to represent a set *S* = {*s*_1_, . . ., *s_m_*} of *m* elements. To insert an element *s_i_*, the bits *B*[*h_j_*(*s_i_*)] for 1 ≤ *j* ≤ *k* are set to 1. Note that the bit remains unchanged when being already set to 1. To check whether an element *x* is in the set *S*, we can check whether the bits *B*[*h_j_*(*x*)] for 1 ≤ *j* ≤ *k* are all 1’s. If and only if they are all equal to 1, *x* is deemed to be an element of *S*. The size *n* of Bloom filter is independent of the size of elements and can be constant, which is very memory-efficient. Nevertheless, the membership query on Bloom filter has false positive but has no false negative. In other words, it is probable to falsely consider an element that actually does not belong to *S* as an element of *S*. In [7], such false positive probability can be obtained as ${\left(1-{\left(1-\frac{1}{n}\right)}^{\mathrm{km}}\right)}^{k}\approx {\left(1-e^{\frac{-\mathrm{km}}{n}}\right)}^{k}$. The optimization between the performance efficiency e.g., array length or hash functions required) of Bloom filter and the false positive probability can be obtained but is beyond the scope of this paper. Note that one of the characteristics of Bloom filter we use in the design of our proposed scheme is that the query result is always correct if the content to be queried is indeed stored in the Bloom filter.

### 2.2. Security Issues in UWSNs

Although UWSNs are studied only recently, many security issues have been investigated. The data survival issue, which aims to prevent the sensed data from being arbitrarily erased by the adversary, is first studied in [1,8]. Due to the fact that the adversary may compromise some sensors in order to enhance its capability of subverting the network functionality, the self-healing techniques are proposed in [9,10] to recover the compromised sensors. We refer the readers to [11] for a comprehensive overview of security issues in UWSNs.

As for the authentication problem in UWSNs, few research efforts are conducted. Since the data sensed within the collection interval should be stored in the sensor for a possibly considerable period of time, in order to provide forward-security (described in Section 3.2), some authentication schemes [3,12] were proposed. Moreover, in [4], two authentication schemes, CoMAC and ExCo, both of which rely on the collaboration among multiple sensors, are presented for collecting authentic sensed data. To our knowledge, CoMAC and ExCo are the only schemes having both forward and backward security (described in Section 3.2). Particularly, in CoMAC, each sensor *j*, after obtaining its sensed data $d_{j}^{r}$ at round *r*, constructs the corresponding authentication tag $z_{j}^{r}$. Then, it immediately sends $〈d_{j}^{r},z_{j}^{r}〉$ to a set of randomly selected sensors. To verify the authenticity of $d_{j}^{r}$, the sink collects all the corresponding authentication tags and checks whether the collected authentication tags can be generated by using the claimed $d_{j}^{r}$. ExCo is different from CoMAC in that the former stores the hash of all the received authentication tags for each sensor while the latter directly keeps the received authentication tags for each sensor.

Indeed, when CoMAC or ExCo is used, forward-security and backward-security can be achieved, enabling the sink to acquire the authentic data from sensors. Unfortunately, the naïve collaboration among sensors used in CoMAC and ExCo incurs many more security problems. For example, the adversary may arbitrarily inject a large number of bogus messages into the network, pretending that it is proceeding with the collaborative authentication procedure albeit the false data will not be accepted by the sink eventually. Such kind of Path-Based DoS (PDoS) attack [5] would deplete the energy of sensors forwarding bogus messages, significantly reducing the network lifetime. Additionally, the compromised sensor can either provide bogus authentication tags to the sink or contaminate the transmitted authentication tags during the collaboration of sensors, forcing the genuine sensed data to be rejected by the sink due to the inconsistency with the authentication tags. The above misbehavior is similar to False-Endorsement DoS (FEDoS) attack [6], and is in common with most of the collaborative security schemes. We also note that, although the resilience of ExCo against sensor compromises is claimed in [4] to be stronger than that of CoMAC that works as a baseline scheme, when the adversary is assumed to be capable of controlling all the compromise sensors and be aware of the target round right after the end of the target round, their resilience will the same. We will describe the above problem in more details in Section 4.1

## 3. Preliminaries

The network and security assumptions used in this paper, as described in the following, are similar to those of the prior works. Nevertheless, the adversary considered in this paper is more realistic and stronger than the ones in the literature.

### 3.1. Network Assumption

In this paper, a UWSN is composed of *n* homogeneous sensors, {*s*_1_, . . ., *s_n_*}, and a sink with mobility. Here, only the sink is assumed to have mobility. The unattended sensors are assumed to be static. These sensors are uniformly scattered over the sensing region to execute the pre-determined functionalities such as data gathering. The underlying network is assumed to be connected such that two arbitrary sensors can communicate with each other through either direct communication or multi-hop path. Time is divided into *collection intervals*, each of which will be further divided into *v* rounds, and can be synchronized by existing techniques [13–15]. In UWSNs, no constant connection between sensors and the sink exists. Instead, the itinerant sink periodically visits the UWSN to collect data at the end of each collection interval. Sensors are scheduled such that, in each round, each sensor obtains exactly one sensed datum. For ease of presentation, we assume that each sensor in each round gets exactly one sensed datum. Note that our proposed method actually can be applied on the case that each sensor can derive multiple sensed data or nothing in a round. After the contact to the data sink, sensors are securely re-initialized. We temporarily assume that the message transmission is reliable, which means that the message can be always be sent to the destination sensor. We will relax this assumption in later sections. In UWSNs, the geographic position of each sensor is known by the sink. This is due to the characteristic of UWSNs that the mobile sink harvests the sensed data by directly visiting the sensors. To do so, the sink must know the position of each sensor in advance. Two possible ways make the sink know the position of each sensor. First, sensors are deterministically deployed one by one by the network owner so that the position information will be passed to the sink by the owner. Second, sensors are randomly deployed, but can be aware of their geographic positions by using techniques such as [16–19] or specific hardwares such as Global Positioning System (GPS).

### 3.2. Security Assumption

**Figure 2.**
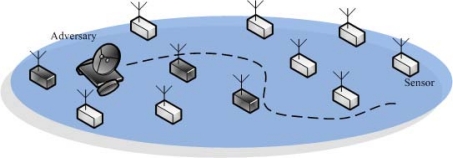
The adversary also roams around the sensing region and compromises the sensors. In this plot, the adversary can compromise one sensor at each round, and three sensors are compromised by the adversary after the first three rounds of a collection interval.

In this paper, the adversary, whose goal is to let the sink accept the fraudulent data that is claimed to be the one sensed by *s_i_* at the target round *r̄*, is considered. The adversary can simultaneously compromise *k* sensors *at each round*. The conceptual illustration is shown in Figure 2. It should be particularly noted that the adversary is assumed to migrate and control different sets of compromised sensors in the literature. We have a stronger adversarial assumption that, once being compromised, the sensor is always under the control of the adversary. The secret information stored in the compromised sensors will therefore be exposed to the adversary. The adversary can launch sensor compromise attack right after the sensor deployment in order to maximize its the possibility of subverting the network functionality. Nevertheless, since each sensor will be reset at the end of each collection interval, we focus on the security issue in a specific collection interval. We also note that the adversary is a global eavesdropper, which means that it can eavesdrop on each message transmitted over the network. In addition, the adversary learns its objective, *s_i_* and *r̄*, at the end of round *r̄*. Note that *s_i_* and *r̄* are unknown to the sink so that the sink cannot apply protection only on specific sensors.

Since the adversary focuses on substituting specific data, with the consideration of the unattended nature of UWSNs, *forward-secure property*, which means that the adversary gains no advantage about the data sensed before the round *r* from the sensor compromised at the round *r*, can be useful in resisting the adversary. Key evolution, in which the key used in round *r* is evolved from the key used in round *r*−1 through the cryptographic hash function, is a simple means to provide forward-security. Nevertheless, forward-secure cryptographic techniques are effective against only *reactive adversary*, which means that the adversary starts compromising sensors after it identifies the target, but is of no use when the *proactive adversary*, which means that the adversary can compromise sensors even before identifying the target, is considered. As a consequence, the *backward-security*, which means that the adversary learns no secret of the next round, is required. An authentication scheme with both forward-security and backward-security is called *key-insulated*, which means that the adversary can learn only the current secret.

## 4. Proposed Method

For completeness, before describing our proposed authentication scheme, we first briefly review the ExCo scheme proposed in [4] and describe its three security defeats in more details in Section 4.1 After that, the basic idea of our proposed Acquire Authentic Data (AAD) method is presented in Section 4.2 Finally, the detailed description of AAD will be presented in Section 4.3

### 4.1. Motivation

In ExCo, at each round *r*, each sensor *j* constructs the MAC $z_{j}^{r}$ of its sensed value $d_{j}^{r}$ and sends $z_{j}^{r}$ to a random subset $R_{j}^{r}$ of sensors. Within the round *r*, each sensor *j* has certain possibility of receiving MACs from the other sensors. If this happens, it temporarily keeps them in the local memory. At the end of round *r*, each sensor *j* constructs and stores the MAC, $H_{j}^{r}=\mathrm{MAC}\left(z_{j}^{r}\left\|z_{q_{1}}^{r}\right\|\cdots \|z_{q_{\phi }}^{r}\right)$, where ‖ denotes the bit-string concatenation, if it receives MACs from the sensors *q*_1_, . . ., *q_ϕ_*. Afterwards, except for $H_{j}^{r}$, all the MACs generated and received in the round *r* are deleted. From the sink point of view, to authenticate the $d_{j}^{r}$, it involves the verification of MAC, $H_{j}^{r}$, and the MACs in $R_{j}^{r}$, where $R_{j}^{r}$ is the set of sensors that send the authentication tags to *s_j_*. Note that the sensors in $R_{j}^{r}$ are called the co-authenticators of *s_j_* at round *r*. For example, if $R_{j}^{r}$ contains *q*_1_, . . ., *q_ϕ_*, then, in addition to the verification of $H_{j}^{r}$, the MACs, $H_{q_{1}}^{r},\ldots ,H_{q_{\phi }}^{r}$ are required to be verified as well.

First, although it is claimed in [4] that ExCo is stronger than CoMAC in terms of sensor compromises, their resilience against sensor compromises is actually the same in practice. Particularly, according to [4], it is claimed that the adversary needs to compromise all the sensors in $\left\{s_{j}\right\}\cup R_{j}^{r}\cup S_{j}^{r}\cup T_{j}^{r}$, where $S_{j}^{r}$ is the set of sensors that receive the authentication tags from *s_j_* and $T_{j}^{r}$ is the set of sensors that send the authentication tags to the sensors in $S_{j}^{r}$, before the round *r*, to successfully subvert the security ExCo provides. Nevertheless, if *r̄* is the target round, the adversary is aware of *r̄* at the round *r̄* + 1, and the adversary only compromises the sensors in $\left\{s_{j}\right\}\cup R_{j}^{r}$, then the adversary can still provide to the sink a counterfeit sensed data without being detected. It can proceed as follows. At first, we assume that, for each round *r*, each compromised sensor keeps the data received in rounds *r* − 1 and *r* in its local memory. As soon as the compromised sensor *s_j_* and compromised sensors in $R_{j}^{\bar{r}}$ are aware of *r̄*, *s_j_* instantly replaces the original sensor reading $d_{j}^{\bar{r}}$ with the value ${\hat{d}}_{j}^{\bar{r}}$ the adversary wants to report to the sink. In addition, the compromised sensors in $R_{j}^{\bar{r}}$ pretend that they also receive ${\hat{d}}_{j}^{\bar{r}}$. Hence, before the end of round *r̄* + 1, *s_j_* can collaborate with the sensors in $R_{j}^{\bar{r}}$ to generate their own MACs that are consistent with each other and are consistent with ${\hat{d}}_{j}^{\bar{r}}$. With these generated MACs, the ${\hat{d}}_{j}^{\bar{r}}$ to be reported to the sink will not be recognized to be counterfeit. Moreover, this is not an attack in an ideal case. Instead, this is a practical attack, because, in practice, the length of each round will not be too short and the adversary can be aware of the target round right after something desired happens. Hence, ExCo is not as strong as [4] claims and has the same security strength as CoMAC.

Second, Exco is vulnerable to PDoS attacks. In particular, after the adversary compromises a few sensors, these compromised sensors can be used to intentionally inject a large amount of useless traffic claimed to be the MACs in ExCo to waste the precious energy of sensors so that the network lifetime will be significantly reduced. Although a simple defense that limits the number of message forwarding at each sensor seems to be helpful in alleviating PDoS attacks, it is indeed useless because the network traffic on each sensor cannot be estimated in advance. As a consequence, if some limits are applied on the number of message forwarding at each sensor, some packets such as transmitted MACs in ExCo will be dropped somewhere on its way to the destination Under the circumstance, the verification will fail and some sensor readings will be regarded as falsified ones because some of the co-authenticators cannot provide the MACs.

**Figure 3.**
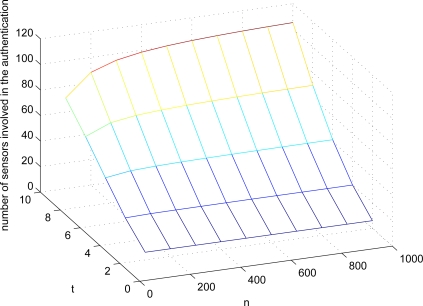
Expected number *ν*(*n, t*) of sensors involved in the authentication of the sensed data of one sensor.

**Figure 4.**
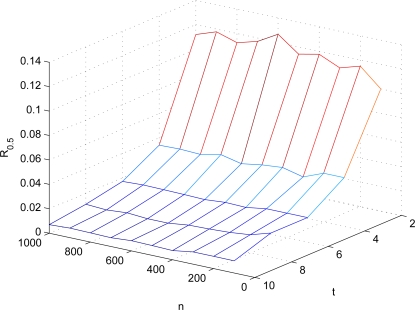
*R*_0.5_’s in different settings.

Third, ExCo is also vulnerable to FEDoS attacks. In fact, this is a common attack if only a simply designed collaboration scheme is used. Specifically, after a sensor is compromised and is inquired by the sink to ask for the MAC used for authenticating the sensed data of the other sensors, it can always reply a random string to the sink. This implies that some data sensed by genuine sensors will be thought of as fake. This is because the MACs involved in a large number of sensors are necessary for authentication, leading to be easily vulnerable to FEDoS attacks. Simply taking the majority of the verification results of MACs from different co-authenticators seems to be helpful in alleviating FEDoS attacks, but it also reduces the resilience against sensor compromises. As a matter of fact, without the proper design, there always exists a dilemma of enhancing the resilience against sensor compromises or enhancing the resilience against FEDoS attacks. In addition, it has been demonstrated [4] that the expected number *ν*(*n, t*) of sensors involved in the authentication of the sensed data of one sensor in ExCo is

(1) $$\nu (n,t)=n\left(1-{\left(\frac{t}{n-1}\right)}^{2}{\left(1-\frac{t}{n-2}\right)}^{t}\right)$$

where $\left|R_{j}^{r}\right|=t$ is the number of co-authenticators. Figure 3 shows different settings of *ν*(*n, t*). Here, we further define a metric *R_r_*, called *FEDoS ratio* of *r*, which denotes the ratio of the number of sensors required to be compromised to the total number of sensors in the network. Note that the parameter *r* means that *r* × 100% of the verifications of sensor readings are affected. FEDoS ratio is used to evaluate and quantify the resilience against FEDoS attacks. According to the *ν*(*n, t*)’s derived as above, *R*_0.5_’s in different settings are shown in Figure 4. It can be observed that when the number of co-authenticators used in ExCo is 2 and approximately 10% of sensors are compromised, over 50% of sensor readings will be regarded as bogus by the sink because of the bogus authentication tags provided by the compromised sensors. When *t* = 4, less than 2% of sensor compromises would result in above 50% of contaminated sensor readings, which will be a disaster in terms of the network security.

### 4.2. Basic Idea

The previous methods are vulnerable to PDoS attack because each sensor can arbitrarily select distant sensors, and sends the authentication tag to the selected sensors. To deal with this problem, our approach is straightforward but effective, *i.e.*, we restrict that the sensor to which the authentication tag is sent cannot be too far away from the sensor sending the authentication tag. Clearly, the effect of PDoS attack can be mitigated because the energy waste due to the message relaying on the intermediate sensors can be reduced. Afterwards, the problem turns to be the design of a proper mechanism that can support the query of proximity information. Here, with the observation that the mobile sink in UWSNs is able to harvest the sensed data because it knows the position of each sensor, different Bloom filters containing different proximity information are stored in different sensors for the query purpose.

On the other hand, the reason that the prior methods suffer from the FEDoS attacks is due to the fact that the compromised sensor can always provide an MAC claimed to be legitimate to the sink. All the sink can do is to verify the data authenticity according to the received MACs. Nevertheless, the received authentication tag could be a random string generated by the compromised sensor, and therefore, the sink declines to accepting the authenticated data. In this respect, our idea is that the MAC used in the authentication procedure should be made in a specific fashion so that the legitimacy of the authentication tag can be verified by the sink before the sink verifies the data authenticity. It should be noted that our defense against FEDoS attacks can also enhance the resilience against sensor compromises. This reveals a particular merit of our method over ExCo.

### 4.3. Proposed Method: AAD

Our proposed AAD scheme consists of five phases: pre-deployment phase, post-deployment phase, sensing phase, receiving/forwarding phase, and verification phase. The pre-deployment phase is executed by the network owner to store necessary materials into the sensors. As its name shows, the post-deployment phase is performed by the sink right after the sensor deployment in order to store the materials required for the authentic data acquisition in the sensors. Sensing phase is executed by each sensor so as to sense data, and distribute the sensed data and the necessary MACs. Once receiving the packet, each sensor executes forwarding phase to forward the received message to the destination sensor. Verification phase is performed by the sink to collect the sensed data and verify their authenticity. The notations used in the following discussion are listed in Table 1. The details of these five phases are described as follows.

**Pre-Deployment Phase.** For each sensor *s_j_*, the sink randomly selects a unique key *K_j_* and a prime number *p_j_*. Note that the prime numbers to be stored in different sensors should be chosen to be distinct. Afterwards, *K_j_* and *p_j_* are stored in the sensor *s_j_*. The sensors are then deployed over the sensing region.

**Post-Deployment Phase.** Let λ be a user-selected parameter, which leverages the security and the energy consumption. The post-deployment phase is used to equip each sensor with a proper Bloom filter for the query of its λ-hop proximity relationship. Here, we say that the sensor *s_j_* has λ-*hop proximity relationship* with the sensor *s_i_* (or, *s_j_* is the λ-hop neighbor of *s_i_*) if the hop distance between *s_j_* and *s_i_* is *within* λ. The post-deployment phase is automatically accomplished when sensors are deterministically deployed by the sink. This is because when deploying the sensor *s_j_*, the sink can also store the Bloom filter ℬ*_j_* containing the λ-hop proximity information of *s_j_* in *s_j_*, given that the position of each sensor is pre-determined by the network owner.

On the other hand, the post-deployment phase will be accomplished within a period of time right after the sensor deployment if sensors are randomly deployed. More specifically, after the sensor deployment, each sensor acquires its geographic position using well-known positioning techniques [16–19], and then reports the acquired position to the sink. Such a reporting should be accompanied by the message authentication code (MAC) with the unique key *K_j_* so that the authenticity of the reported position can be guaranteed. Note that, the falsified position information could be injected by the adversary. Nevertheless, the falsified position information will be found by the sink because the sink will move to the position, trying to collect the sensed data. Thus, if falsified positions are injected by the adversary, they will be easily detected. After receiving each sensor’s position information, the sink first checks its authenticity by examining whether it has the correct MAC. In particular, when the received message is 〈*ℓ_j_, ħ*〉, if *ħ* is equal to *h*_*K*_*j*__(*ℓ_j_*), then *ℓ_j_* is regarded as the position of *s_j_*. With the geographic position of each sensor, the sink can construct the *network graph*, wherein the vertices denote the sensors and the edge between two vertices exists if the corresponding sensors can communicate with each other directly, according to the communication range predefined on each sensor. With the constructed network graph, the sink can be aware of the λ-hop proximity relationship of each sensor. Now, before the sensors start to sense data, the mobile sink starts its first itinerary over the network. For the contact of each sensor *s_j_*, the sink stores a Bloom filter, ℬ*_j_*, containing the λ-hop proximity information of *s_j_*, in *s_j_*. ℬ*_j_* can be constructed by the sink according to the topology of the network graph. Specifically, for each *s_i_* that has λ-hop proximity relationship with *s_j_*, the sink embeds *s_j_*‖*s_i_* into ℬ*_j_*. Note that although the number of λ-hop neighbors of *s_j_* could be different for each *s_j_*, the size of Bloom filter used is chosen to be the same.

**Figure 5.**
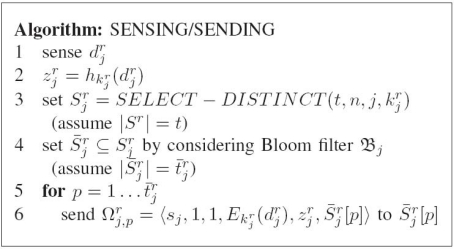
The algorithm of sensing phase.

**Sensing Phase**. The description of sensing phase is shown in Figure 5. The sensing phase is executed by each sensor at each round. The sensor *s_j_* constructs the corresponding MAC, $z_{j}^{r}=h_{k_{j}^{r}}\left(d_{j}^{r}\right)$, after it has the sensed data $d_{j}^{r}$ at round *r*. If the sensed data and corresponding MAC are simply stored in its local memory, once the sensor is compromised by the adversary, all the security materials will be exposed to the adversary, and $d_{j}^{r}$ and $z_{j}^{r}$ can be arbitrarily generated so that the security breach will occur. Thus, in the sensing phase, the sensed data and its corresponding MACs of a sensor will be distributed over some sensors randomly chosen from its λ-hop neighbors. Specifically, a subset $S_{j}^{r}$ of *t* sensors is first randomly sampled from {*s*_1_, . . ., *s_n_*} (line 3 in Figure 5). *SELECT* − *DISTINCT* (*t*, *n*, *j*, $k_{j}^{r}$) is a function randomly generating *t* sensor IDs from {*s*_1_, . . ., *s_n_*} \ {*s_j_*}. It can be implemented by using the hash function whose output is of length ⌈log *n*⌉ bits. Then, a subset ${\bar{S}}_{j}^{r}$ of $S_{j}^{r}$ is constructed by choosing the sensors in $S_{j}^{r}$ whose hop distance to *s_j_* is within the pre-determined threshold λ. Here, each sensor *s_j_* uses the Bloom filter ℬ*_j_* constructed in post-deployment phase to check the hop distances to the randomly selected sensors in ${\bar{S}}_{j}^{r}$. To construct ${\bar{S}}_{j}^{r}$, what each sensor *s_j_* needs to do is to check whether the *s_j_* itself has λ-hop proximity relationship with the generated sensors in $S_{j}^{r}$. For example, for $s_{i}\in S_{j}^{r}$, the sensor issues *s_j_*‖*s_i_* to query ℬ*_j_*. We can know that $s_{i}\in {\bar{S}}_{j}^{r}$ if the query result is positive and $s_{i}\notin {\bar{S}}_{j}^{r}$ otherwise (line 4 in Figure 5). Afterwards, the MAC, $z_{j}^{r}$, is transmitted to the sensors in ${\bar{S}}_{j}^{r}$. Note that ${\bar{S}}_{j}^{r}={\bar{t}}_{j}^{r}$ may vary as *j* and *r* vary. In general, ${\bar{t}}_{j}^{r}$ is not a constant. Nonetheless, for ease of the explanation, ${\bar{t}}_{j}^{r}$ is described as if it is a constant. In the following, the term “authentication tag of *s_j_*” is used to denote $z_{j}^{r}$ and the term “co-authenticator of *s_j_* at round *r*” is used to denote the sensors in ${\bar{S}}_{j}^{r}$. Then, *s_j_*, according to $d_{j}^{r}$, derives $E_{k_{j}^{r}}\left(d_{j}^{r}\right)$ and $z_{j}^{r}$ to guarantee the data privacy and authenticity, respectively. Lastly, *s_j_* sends $\Omega_{j}^{r}$ to the sensors in ${\bar{S}}_{j}^{r}$ whose second and third fields are always fixed to be 1 (lines 5 and 6 in Figure 5).

**Figure 6.**
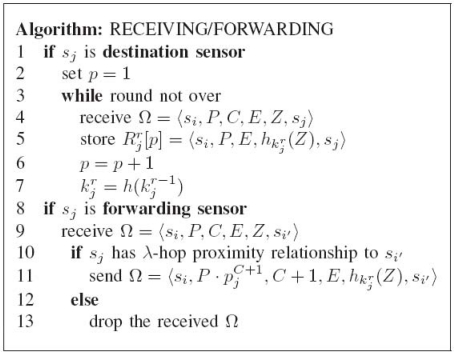
The algorithm of receiving/forwarding phase.

**Receiving/Forwarding Phase.** The algorithm of this phase is described in Figure 6. This phase is executed by each sensor when a message is received. According to the role it acts, each sensor executes different tasks (lines 1∼7 in Figure 6); the receiving task will be executed if the received message is destined to itself, and the forwarding task (lines 8∼13 in Figure 6) is performed otherwise. Assume that *s_j_* is the forwarding sensor and receives Ω = 〈*s_i_, P, C, E, Z, s_i_*_′_〉 (line 9 in Figure 6). At first, *s_j_* checks if it has λ-hop proximity relationship to the destination sensor *s_i_*_′_ (lines 9∼13 in Figure 6). *s_j_* proceeds the procedures if *s_j_* has λ-hop proximity relationship to the destination sensor *s_i_*_′_ and drops the received Ω otherwise. *s_j_* then adds the existence evidence of itself on the forwarding path on Ω so that the sink can check whether the received authentication tag passes through exactly those sensors it should pass. To achieve this goal, *s_j_* applies the keyed hash function with its secret key $k_{j}^{r}$ on *Z* to have $h_{k_{j}^{r}}\left(Z\right)$. Moreover, to enable the sink to accomplish the verification, the information about the forwarding path should be included in Ω; it is the usage of *P* and *C* in Ω. *C* means that *s_j_* is on the (*C* + 1)-th sensor of the forwarding path and *P* can be used to extract all the sensor IDs on the forwarding path (The details will be stated in the description of the verification phase later). More specifically, after receiving Ω, *s_j_* increases *C* by one and multiplies *P* with $p_{j}^{C+1}$, where *p_j_* is the prime number stored in *s_j_* in pre-deployment phase. Afterwards, the packet $〈s_{i},P\cdot p_{j}^{C+1},C+1,E,h_{k_{j}^{r}}\left(Z\right),s_{i^{\prime }}〉$ will be forwarded to the next sensor on the forwarding path. Note that the selection of the next sensor depends on the routing protocol the underlying network uses and is not the focus of this paper. Nevertheless, due to the fact that each sensor knows its position, geographic routing [20] is a reasonable choice.

On the other hand, assume that *s_j_* is the destination sensor and receives Ω = 〈*s_i_, P, C, E, Z, s_j_*〉 (line 4 in Figure 6). Under this situation, *s_j_* simply extracts 〈*s_i_, P, E, Z*〉 from Ω and then stores it into an ordered set $R_{j}^{r}$ (line 5 in Figure 6). At the end of the round *r*, the key $k_{j}^{r}$ will be evolved to the key $k_{j}^{r+1}$ of the next round *r* + 1 using the publicly-known hash function *h* (line 7 in Figure 6).

**Figure 7.**
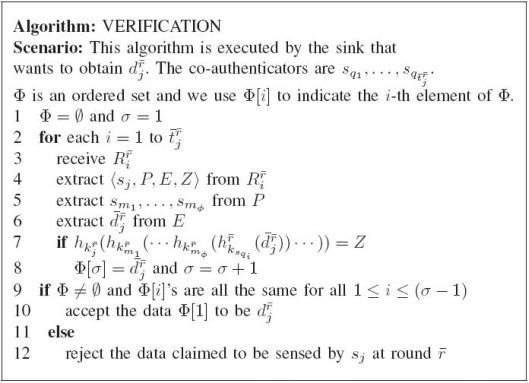
The algorithm of verification procedure.

**Verification Phase.** The algorithm describing the verification phase is shown in Figure 7. Assume that the sink would like to obtain the data $d_{j}^{\bar{r}}$ sensed by sensor *s_j_* at the round *r̄*. What the sink should do is to perform the verification phase. Since the initial key of *s_j_* is given by the sink, the key $k_{j}^{\bar{r}}$ will be known by the sink. Thus, the co-authenticators of *s_j_* at *r̄* will also be known by the sink. This prevents the traces from being deleted by the adversary. The strategy of the sink is to move to the positions near those co-authenticators to collect $d_{j}^{\bar{r}}$, acquiring the proper authentication tags. More specifically, assume that the co-authenticators of *s_j_* are $s_{q_{1}},\ldots ,s_{q_{{\bar{t}}_{j}^{\bar{r}}}},s_{q_{1}},\ldots ,s_{q_{{\bar{t}}_{j}^{\bar{r}}}}\in \{1,\ldots n\}\backslash \{j\}$. For each $s_{q_{i}},i\in \left[1,{\bar{t}}_{j}^{\bar{r}}\right]$, the sink acquires $R_{i}^{\bar{r}}$ and extracts 〈*s_j_, P, E, Z*〉 (lines 3∼4 in Figure 7). Here, 〈*s_j_, P, E, Z*〉’s extracted from different co-authenticators will not be the same in essence. We, however, omit the necessary subscript and superscript of *P, E*, and *Z* without the ambiguity for convenience. From the acquired 〈*s_j_*, *P*, *E*, *Z*〉, with the proper key, the sink can decrypt to obtain the ${\bar{d}}_{j}^{\bar{r}}$ (line 6 in Figure 7), which is claimed by *s_q_i__* to be the data sensed by *s_j_* at round *r̄*. Based on the decrypted ${\bar{d}}_{j}^{\bar{r}}$, the sink would try to verify if the authentication tag can be regenerated to match the authentication tag extracted from $R_{i}^{\bar{r}}$. Here, to reproduce the authentication tag, the sink needs to obtain the sensor IDs on the forwarding path of Ω in the correct order. Then, since the sink knows the keys of all the sensors on the forwarding path, if we can know that *s_j_* → *s*_*m*_1__ → *s*_*m*_2__ → ⋯ → *s*_*m*_*ϕ*__ is the correct order of sensors on the forwarding path connecting *s_j_* and *s*_*q*_*i*__, the sink can construct $h_{k_{j}^{\bar{r}}}\left(h_{k_{m_{1}}^{\bar{r}}}\left(\cdots h_{k_{m_{\varphi }}^{\bar{r}}}\left(h_{k_{s_{q_{i}}}}^{\bar{r}}\left({\bar{d}}_{j}^{\bar{r}}\right)\right)\cdots \right)\right)$, where *s*_*m*_1__, . . ., *s_m_ϕ__* are the sensors on the route from *s_j_* to *s*_*q*_*i*__, and see if it is equal to the authentication tag extracted from 〈*s_j_, P, E, Z*〉. To know *s*_*m*_1__, . . ., *s_m_ϕ__* (line 5 in Figure 7), the sink simply performs prime number factorization of *P*, obtaining ${\hat{p}}_{x_{1}}^{1},{\hat{p}}_{x_{2}}^{2},\ldots ,{\hat{p}}_{x_{\phi }}^{\phi }$, where *ϕ* is the length of the path connecting the sensor *s_j_* and its co-authenticator being examined by the sink. Here, we should note that *ϕ* varies when different co-authenticators are considered. Nonetheless, for ease of explanation, we also omit the necessary subscript and superscript. Then, the sink knows that *s*_*m*_*i*__ = *s*_*x*_*i*__ for all 1 ≤ *i* ≤ *ϕ*. Note that after the factorization of *P*, if *P* cannot be represented as the form of ${\hat{p}}_{x_{1}}^{1},{\hat{p}}_{x_{2}}^{2},\ldots ,{\hat{p}}_{x_{\phi }}^{\phi }$, then the authentication material provided by the co-authenticator being examined will be ignored. The authentication material 〈*s_j_, P, E, Z*〉 extracted from *s*_*q*_*i*__ is dropped by the sink if the regenerated authentication tag does not match the authentication tag in 〈*s_j_, P, E, Z*〉 (line 7 in Figure 7). The decrypted data ${\bar{d}}_{j}^{\bar{r}}$ is stored if $h_{k_{j}^{\bar{r}}}\left(h_{k_{m_{1}}^{\bar{r}}}\left(\cdots h_{k_{m_{\varphi }}^{\bar{r}}}\left(h_{k_{s_{q_{i}}}}^{\bar{r}}\left({\bar{d}}_{j}^{\bar{r}}\right)\right)\cdots \right)\right)$ is equal to the authentication tag extracted from $R_{i}^{\bar{r}}$. After the sink accomplishes the above procedures, if no authentication tag can be successfully verified, then all the data claimed to be the data sensed by *s_j_* at round *r̄* are dropped. Assume that the sink accomplishes the above procedures and at least one authentication tag of co-authenticators can be successfully verified. If and only if ${\bar{d}}_{j}^{\bar{r}}$ extracted from the authentication materials sent from co-authenticators are all the same, ${\bar{d}}_{j}^{\bar{r}}$ are regarded as genuine (lines 9*∼*12 in Figure 7).

**Figure 8.**
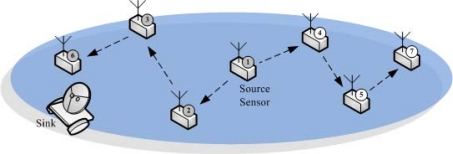
The network topology in the example.

**Example.** Assume that λ = 3 and a specific round *r* is considered. For the sensor *s*_1_, we have the assumption that $d_{1}^{r}=5$, $k_{1}^{r}=7$, $E_{k_{1}^{r}}\left(d_{1}^{r}\right)=E_{7}(5)=10$, and $z_{1}^{r}=h_{7}(5)=8$. In addition, we assume that after the execution of *SELECT* − *DISTINCT* and the checking procedure on ℬ_*j*_, ${\bar{S}}_{r}^{1}=\left\{s_{6},s_{7}\right\}$. Suppose that the shortest path connecting *s*_1_ and *s*_6_ is *s*_1_ → *s*_2_ → *s*_3_ → *s*_6_, and the shortest path connecting *s*_1_ and *s*_7_ is *s*_1_ → *s*_4_ → *s*_5_ → *s*_7_. The network topology in this example is shown in Figure 8. Then, since *s*_1_ has to transmit *E*_7_(5) and $z_{1}^{r}$ to *s*_6_ and *s*_7_, *s*_1_ will send $\Omega_{1}^{r}=〈s_{1},1,1,E_{k_{1}^{r}}(5),z_{1}^{r},s_{6}〉$ and $\Omega_{1}^{r}=〈s_{1},1,1,E_{k_{1}^{r}}(5),z_{1}^{r},s_{7}〉$ to *s*_2_ and *s*_3_, respectively. Since the sensors on these two paths work similarly, we only discuss the path *s*_1_ → *s*_2_ → *s*_3_ → *s*_6_. When *s*_2_ receives 〈*s*_1_, 1, 1, 10, 8, *s*_6_〉, it first check its λ-hop proximity relationship to *s*_1_. Here, since we assume that *λ* = 3, the check can be passed. Thus, assuming that *p*_2_ = 3, *s*_2_ forwards 〈*s*_1_, 9, 2, 10, $h_{k_{2}^{r}}(8)$, *s*_6_〉 to *s*_3_. Similarly, if we assume that *p*_3_ = 7, after receiving 〈*s*_1_, 9, 2, 10, $h_{k_{2}^{r}}(8)$, *s*_6_〉, *s*_3_ sends 〈*s*_1_, 3087, 3, 10, $h_{k_{3}^{r}}\left(h_{k_{2}^{r}}(8)\right)$, *s*_6_〉 to *s*_6_. When *s*_6_ receives 〈*s*_1_, 3087, 3, 10, $h_{k_{3}^{r}}\left(h_{k_{2}^{r}}(8)\right)$, *s*_6_〉, it stores 〈_s__1_, 3087, 10, $h_{k_{6}^{r}}\left(h_{k_{3}^{r}}\left(h_{k_{2}^{r}}(8)\right)\right)$, *s*_6_〉 in its local memory.

Now consider that the sink wants to obtain $d_{1}^{r}$. In our method, the sink has to perform two verifications on *s*_6_ and *s*_7_. Basically, because the verification performed on *s*_6_ is the same as the one performed on *s*_7_, we only describe the one performed on *s*_6_. From *s*_6_, the sink can obtain 〈*s*_1_, 3087, 10, $h_{k_{6}^{r}}\left(h_{k_{3}^{r}}\left(h_{k_{2}^{r}}(8)\right)\right)$〉. After performing the prime number factorization of the value 3087, the sink can know that 3087 = 3^2^ × 7^3^, which means that the second hop sensor and the third hop sensor on the path connecting *s*_1_ and *s*_6_ are *s*_2_ and *s*_3_, respectively. The sink also extracts ${\bar{d}}_{j}^{\bar{r}}=5$ from $E_{k_{1}^{r}}(5)=10$. Then, the sink checks if $h_{k_{6}^{r}}\left(h_{k_{3}^{r}}\left(h_{k_{2}^{r}}\left(8\right)\right)\right)$ is equal to the value obtained by sequentially applying three keyed hash functions $h_{k_{2}^{r}}\left(\cdot \right)$, $h_{k_{3}^{r}}\left(\cdot \right)$, and $h_{k_{6}^{r}}\left(\cdot \right)$ on $z_{1}^{r}=8$. ${\bar{d}}_{j}^{\bar{r}}$ will be temporarily stored if the above check is passed and dropped otherwise. Finally, when all the co-authenticators are visited, the sink checks the consistency of ${\bar{d}}_{j}^{\bar{r}}$’s obtained from different co-authenticators. Note that for the notational simplicity, we do not put additional subscript on ${\bar{d}}_{j}^{\bar{r}}$ to distinguish different ${\bar{d}}_{j}^{\bar{r}}$’s obtained from different co-authenticators. ${\bar{d}}_{j}^{\bar{r}}$ is deemed to be genuine if they are consistent and bogus otherwise.

## 5. Performance and Security Analysis

In the section, the performance and security of AAD will be evaluated. Recall that at each round *r*, the adversary is able to compromise the set *C_r_* of *k* sensors. Without the loss of generality, we assume in the subsequent discussion that *C_r_* ∩ *C_r_*_′_ = ∅ if *r* ≠ *r′*.

### 5.1. Security Analysis

In what follows, the resilience of AAD against sensor compromises, PDoS attack and FEDoS attack will be described, respectively.

**Resilience Against Sensor Compromises**. Let ${\hat{d}}_{j}^{\bar{r}}$ be the counterfeit data the adversary constructs to substitute $d_{j}^{\bar{r}}$. For the adversary, to successfully deceive the sink into accepting the bogus ${\hat{d}}_{j}^{\bar{r}}$, it needs to compromise all the sensors in ${\bar{S}}_{j}^{\bar{r}}$ and ${ℰ}_{{\bar{S}}_{j}^{\bar{r}}}$, where ${ℰ}_{{\bar{S}}_{j}^{\bar{r}}}$ denotes the set of all the sensors on the route from *s_j_* to the sensors in ${\bar{S}}_{j}^{\bar{r}}$, before the target round *r̄*. In other words,

(2) $${\bar{S}}_{j}^{\bar{r}}\cup {ℰ}_{{\bar{S}}_{j}^{\bar{r}}}\subseteq \left\{C_{1},\ldots ,C_{\bar{r}}\right\}$$

Compromising the sensors in ${\bar{S}}_{j}^{\bar{r}}$ is used to substitute the authentication tag corresponding to ${\hat{d}}_{j}^{\bar{r}}$ while compromising the sensors in ${ℰ}_{{\bar{S}}_{j}^{\bar{r}}}$ aims to know the keys stored in the sensors in ${ℰ}_{{\bar{S}}_{j}^{\bar{r}}}$ so that the legitimacy of the bogus authentication tag can also be counterfeited.

To evaluate the resilience of AAD against the sensor compromises, we have to estimate the number of sensors involved in the authentication procedure of one datum in AAD. Let |*N_σ_*| be the number of sensors that is exactly *σ*-hop away from a specific sensor in a randomly deployed network. Before starting to estimate the number of sensors involved in the authentication procedure of one datum in AAD, we first need to know how to calculate |*N_σ_*|, 1 ≤ *σ* ≤ λ. Obviously, |*N_σ_*| is dependent on the size of the sensing region, the number *n* of sensors, and the communication range *R* of each sensor. Although $\left|N_{1}\right|=\pi R^{2}\frac{n}{A}-1$ can be easily obtained, there is no explicit formula for the expression of *N_σ_* for *σ* ≥ 2. An approximation of |*N_σ_*| is calculated in [21]. Although the calculation of *N*_1_ is straightforward and the calculation of *N*_2_ is not difficult, calculating *N*_3_ is quite difficult. Considering two sensors *a* and *b* with communication range being *r*. There could be the case that *b* lies in the communication disk centered at *a* with the radius 3*r* but *a* cannot reach *b via* the exactly 3-hop communication. Even if *b* which lies in the communication disk centered at *a* with the radius 3*r* can reach *a*, we have to make sure whether they can communicate with each other *via* 3-hop communication or they can communicate with each other *via* 2 or 1-hop communication. Simultaneously considering all these kinds of possibilities is an extremely difficult task. To the best of our knowledge, no exact formula has been proposed to deal with the calculation of *N_σ_*. As [21] indicates, *N_σ_* is well approximated by using their proposed approximation formula. In our AAD scheme, for the energy conservation, *λ* is usually not too large, which means that we do not need to consider *N_σ_* for large *σ*. Thus, although the approximation formula of *N_σ_* gradually deviates from the true *N_σ_* as *σ* increases, it is still sufficient for our use. Therefore, in the following discussion, we refer to [21] for the derivation of |*N_σ_*|, 2 ≤ *σ* ≤ *λ*. For 1 ≤ *σ* ≤ *λ*, the probability of randomly selecting a sensor whose hop distance to the sensor *s_j_* is exactly *σ* can be expressed as $\frac{\left|N_{\sigma }\right|}{n}$. Therefore, the probability of randomly selecting a sensor whose hop distance to *s_j_* is within *λ* is $\frac{\sum_{\sigma =1}^{\lambda }\left|N_{\sigma }\right|}{n}$, and the expected number of randomly chosen sensors whose hop distance to *s_j_* is within *σ* is $\frac{\sum_{\sigma =1}^{\lambda }\left|N_{\sigma }\right|}{n}\cdot t$ when *t* sensors are randomly chosen. These $\frac{\sum_{\sigma =1}^{\lambda }\left|N_{\sigma }\right|}{n}\cdot t$ sensors exactly constitute the set of sensors in ${\bar{S}}_{j}^{\bar{r}}$. Recall that one of the characteristics of Bloom filter is that the query result is always correct if the content to be queried is indeed stored in the Bloom filter. Thus, we have ${\bar{t}}_{j}^{\bar{r}}=\frac{\sum_{\sigma =1}^{\lambda }\left|N_{\sigma }\right|}{n}\cdot t$. On the other hand, all the sensors on the route to the sensors in ${\bar{S}}_{j}^{\bar{r}}$ also contribute to the authentication procedure. As the number of these sensors is difficult to be precisely calculated, we will approximate it by assuming that the routes to the selected sensors in ${\bar{S}}_{j}^{\bar{r}}$ are non-overlapping. For each sensor *s_i_* that is exactly *σ*-hop away from *s_j_*, *σ* − 1 sensors lie on the route between *s_j_* and *s_i_*. Thus, $\sum_{\sigma =1}^{\lambda }\frac{\left|N_{\sigma }\right|}{n}t(\sigma -1)$ sensors are involved when $\frac{\sum_{\sigma =1}^{\lambda }\left|N_{\sigma }\right|}{n}t$ sensors are in ${\bar{S}}_{j}^{\bar{r}}$. It implies that, $\left|{\bar{S}}_{j}^{\bar{r}}\cup {ℰ}_{{\bar{S}}_{j}^{\bar{r}}}\right|$ can be upper bounded by:

(3) $$\begin{array}{l} \;\;\;\;\;\frac{\left|N_{1}\right|}{n}t(1+0)+\cdots +\frac{\left|N_{\lambda }\right|}{n}t(1+\lambda -1)\\ =\sum_{\sigma =1}^{\lambda }\frac{\left|N_{\sigma }\right|}{n}t\sigma \end{array}$$

Because of our assumption that the routes from *s_j_* to the sensors in ${\bar{S}}_{j}^{\bar{r}}$ are non-overlapped, this value overestimates $\left|{\bar{S}}_{j}^{\bar{r}}\cup {ℰ}_{{\bar{S}}_{j}^{\bar{r}}}\right|$ and, thus, can only work as the upper bound of $\left|{\bar{S}}_{j}^{\bar{r}}\cup {ℰ}_{{\bar{S}}_{j}^{\bar{r}}}\right|$. In practice, the approximation of $\left|{\bar{S}}_{j}^{\bar{r}}\cup {ℰ}_{{\bar{S}}_{j}^{\bar{r}}}\right|$ in Equation (3) is quite accurate when the ratio $\frac{{\bar{t}}_{j}^{r}}{\eta }$, where *η* denotes the average number of one-hop neighbors for a sensor, is sufficiently small. Nonetheless, since we know that the fewer the sensors involved in the authentication procedure, the lower the resilience against sensor compromises, even Equation (3) provides a good approximation of $\left|{\bar{S}}_{j}^{\bar{r}}\cup {ℰ}_{{\bar{S}}_{j}^{\bar{r}}}\right|$ in most cases and can be used to estimate the upper bound of communication overhead in Section 5.2, we still need a lower bound of $\left|{\bar{S}}_{j}^{\bar{r}}\cup {ℰ}_{{\bar{S}}_{j}^{\bar{r}}}\right|$ to derive the lower bound of the resilience of AAD against sensor compromises. In essence, even if the setting of ${\bar{t}}_{j}^{r}\geq 1$ is used, the lower bound of $\left|{\bar{S}}_{j}^{\bar{r}}\cup {ℰ}_{{\bar{S}}_{j}^{\bar{r}}}\right|$ can be obtained according to the setting of ${\bar{t}}_{j}^{r}=1$. By the similar calculation as in Equation (3), we can know that $\left|{\bar{S}}_{j}^{\bar{r}}\cup {ℰ}_{{\bar{S}}_{j}^{\bar{r}}}\right|\geq L(\lambda ,n)$, where

(4) $$L(\lambda ,n)=\sum_{\sigma =1}^{\lambda }\frac{\left|N_{\sigma }\right|}{n}\sigma$$

Define *P_ADV_* as the probability that the event described in Equation (2) happens. Thus, for the adversary that always randomly compromises the sensors over the whole network, the upper bound of probability *P_ADV_* can be obtained as:

(5) $$\{\begin{array}{ll} \frac{\left(\begin{array}{c} n-L(\lambda ,n)\\ k\bar{r}-L(\lambda ,n) \end{array}\right)}{\left(\begin{array}{c} n\\ k\bar{r} \end{array}\right)} & \text{if}\;k\bar{r}\;\;\leq \;\;n\\ 1 & \text{if}\;k\bar{r}\;\;>\;\;n \end{array}$$

With *kr̄* > *n*, it means that all the sensors are compromised and the whole network is in the complete control of the adversary. On the other hand, with *kr̄* ≤ *n*, there are $\left(\begin{array}{c} n\\ k\bar{r} \end{array}\right)$ ways to compromise the sensors in the networks and at least $\sum_{\sigma =1}^{\lambda }\frac{\left|N_{\sigma }\right|}{n}\sigma$ specific sensors should be included in the set {*C*_1_, . . ., *C_r̄_*} of compromised sensors. As a result, the upper bound of *P_ADV_* defined in Equation (5) holds. Here, it should be noted that instead of compromising random sensors over the network, the adversary that is aware of the application of AAD on the network may intentionally compromise the sensors in the nearby region of *s_j_* so as to increase its success probability *P_ADV_* . Nevertheless, this strategy is infeasible for the adversary since the target sensor *s_j_* and the target round *r̄* are known only after the end of the target round. Thus, without the prior knowledge of the target sensor, in general, what the adversary can do is only to randomly compromise the sensors over the network. Define *P_AAD_* as the *survival probability*, the probability that the data $d_{j}^{\bar{r}}$ remain unforged can be calculated as:

(6) $$P_{\mathrm{AAD}}=1-P_{\mathrm{ADV}}$$

Because of the upper bound of *P_ADV_* in Equation (5), the lower bound of *P_AAD_* can be represented as:

(7) $$\{\begin{array}{ll} 1-\frac{\left(\begin{array}{c} n-L(\lambda ,n)\\ k\bar{r}-L(\lambda ,n) \end{array}\right)}{\left(\begin{array}{c} n\\ k\bar{r} \end{array}\right)} & \text{if}\;k\bar{r}\;\;\leq \;\;n\\ 0 & \text{if}\;k\bar{r}\;\;>\;\;n \end{array}$$

**Figure 9.**
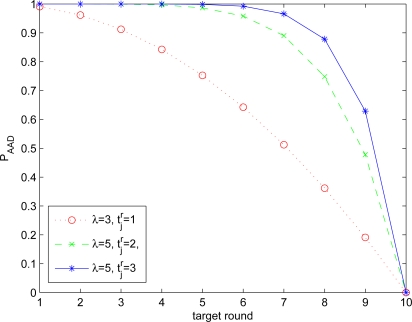
*P_AAD_* in the setting of *n* = 100, *k* = 10, and |*N*_1_| = 10.

*P_AAD_*’s in different settings are shown in Figure 9. Note that the dotted line represents the lower bound of *P_AAD_* in the setting of *n* = 100, *k* = 10, and |*N*_1_| = 10, irrespective of the parameter *t* used in AAD. Although the dashed line and solid line act as the upper bound of *P_AAD_* in the setting of *n* = 100, *k* = 10, and |*N*_1_| = 10. They, however, are pretty accurate approximations of *P_AAD_* in a relatively dense network. We can observe that the curve of survival probabilities generated by the use of AAD with the parameters ${\bar{t}}_{j}^{r}=3$ and *λ* = 5 is close to that in [4] with 15 co-authenticators used in their proposed protocol. Therefore, we can also claim that, when the similar level of security is required, the communication overhead incurred by AAD is lower than that incurred by prior works.

**Figure 10.**
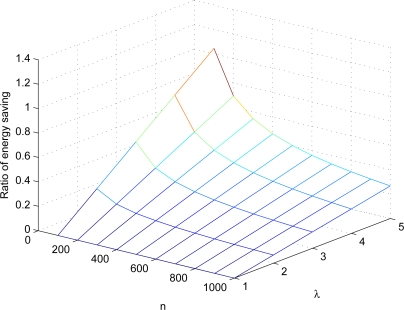
The ratio of energy saving of our AAD method over ExCo in different settings.

**Resilience Against PDoS attacks.** In general, the adversary is always able to launch PDoS attacks. In other words, PDoS attacks can only be mitigated, but not eliminated. The evaluation method similar to the ones used in [22–25] is conducted here to demonstrate the superiority of AAD over CoMAC and ExCo in terms of the resilience against PDoS attacks. Recall that the resilience of CoMAC and ExCo are the same. Let *L_AAD_* and *L_ExCo_* be the bit-length of the authentication material of AAD and ExCo, respectively. Assume that a MAC is of length 128 bits and an encrypted data is also of length 128 bits. In AAD, the field representing the counter *C* is assumed to be of length 8 bits because the setting of *λ* ≤ 2^8^ − 1 is sufficient in most cases. The field representing the aggregated prime number *P* is assumed to have 32 bits. Assume that in total *n* sensors are deployed. Consequently, *L_AAD_* can be calculated as $128+128+32+8+32+2\sqrt{n}=328+2\sqrt{n}$ bits. On the other hand, since the authentication material only contains the authentication tag, the size of *L_ExCo_* is $128+2\sqrt{n}$ bits. For a flat network whose *n* sensors are uniformly distributed, the average hop distance between arbitrary two sensors is $\sqrt{n}$ [26]. In the following evaluation, it is assumed that the compromised sensor sends bogus message to a random sensor, instead of sending to the most distant sensors. Also note that such evaluation is advantageous to the calculation of ExCo’s energy consumption but is disadvantageous to the calculation of ours. Nevertheless, such evaluation will imply the lower bound of the energy saving of our AAD over ExCo. We can know from [23] that when telosB motes [27] are used, the energy for receiving one bit requires 0.2707*μ*J and the energy for transmitting one bit requires 0.2505*μ*J. In ExCo, the energy *E_ExCo_* wasted by the forwarding of one single bogus message in the PDoS attack is therefore $0.5212\cdot (\sqrt{n}-1)\cdot L_{\mathrm{ExCo}}=0.5212\cdot (\sqrt{n}-1)\cdot \left(128+2\sqrt{n}\right)\mu J$. In AAD, the energy *E_AAD_* wasted by the forwarding of one single bogus message in the PDoS attack is therefore $0.5212\cdot (\lambda -1)\cdot L_{\mathrm{AAD}}=0.5212\cdot (\lambda -1)\cdot \left(328+2\sqrt{n}\right)\mu J$. Hence, the ratio of energy saving of our AAD method to ExCo can be expressed as $\frac{0.5212\cdot (\lambda -1)\cdot \left(328+2\sqrt{n}\right)}{0.5212\cdot (\sqrt{n}-1)\cdot \left(128+2\sqrt{n}\right)}$ and is depicted in Figure 10. Since the ratio less than one means that the energy consumed by AAD is lower than that consumed by ExCo, we can observe in Figure 10 that in most cases the energy consumption of AAD is lower than that of ExCo. Note that the energy calculation is based on the energy consumed by the message transmission from the source to the destination. Since the adversary can always launch PDoS attacks, the calculation is independent of the parameter *t*. The energy saving of AAD is due to the use of *λ* in restricting the number of hops used to relay the messages.

**Figure 11.**
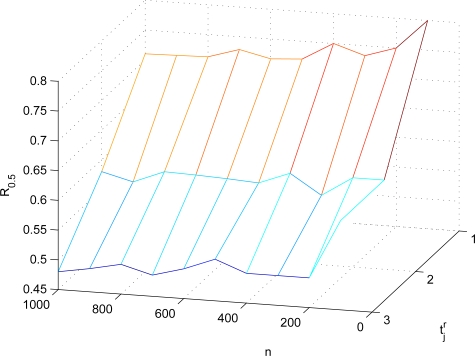
*R*_0.5_’s of AAD in different settings (|*N*_1_| = 5).

**Figure 12.**
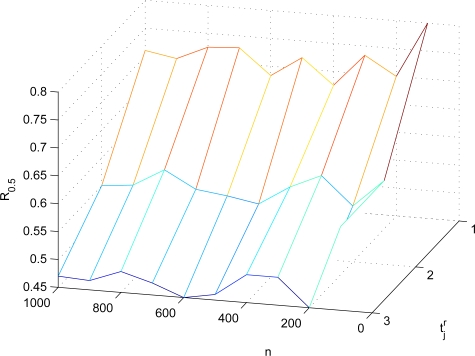
*R*_0.5_’s of AAD in different settings (|*N*_1_| = 10).

**Resilience Against FEDoS attacks.** Recall that FEDoS attacks aim to deceive the sink into rejecting the genuine sensor readings by providing false authentication tags. FEDoS attacks easily work because what the sink can trust is only the authentication tag the co-authenticators provide. Therefore, if one of co-authenticators is compromised, the false authentication tag generated by it makes FEDoS attack successful. Nevertheless, in AAD, for a compromised sensor that happens to be a co-authenticator of the data of the sink’s interest, it cannot simply generate and then provide a random bit string acting as the authentication tag to the sink. Instead, together with the claimed authentication tag, some additional information should be accompanied so that before accepting the received bit string as the genuine authentication tag, the sink can check the legitimacy of the claimed authentication tag. Specifically, *P* and *E* serve for this purpose. From the algorithm of the verification phase summarized in Figure 7, it can be observed that for the route to a specific co-authenticator, unless the keys of all the sensors on the route are known by the adversary and the corresponding co-authenticator is compromised prior to the round *r̄*, the authentication tag cannot be counterfeited. We have an interesting observation that, in the case where ${\bar{t}}_{j}^{\bar{r}}=1$, successfully launching FEDoS attacks is as difficult as successfully replacing the data on the target sensor at the target round because they all need to compromise the sensors in ${\bar{S}}_{j}^{\bar{r}}$ and the sensors on the route from the target sensor to the sensor in ${\bar{S}}_{j}^{\bar{r}}$. Here, we also employ FEDoS ratio to evaluate the resilience of AAD against FEDoS attacks. When the average number |*N*_1_| of the one-hop number of the sensor in a network is 5, *R*_0.5_’s of AAD in different settings are shown in Figure 11. When the average number |*N*_1_| of the one-hop number of the sensor in a network is 10, *R*_0.5_’s of AAD in different settings are shown in Figure 12. It can be known from Figures 11 and 12 that the resilience of AAD against FEDoS attacks is independent of the network topology such as the sensor density and the number of sensors. In addition, it can be observed that, as ${\bar{t}}_{j}^{r}$ increases, *R*_0.5_ will be decreased. The reason for this phenomenon is that, the increase of ${\bar{t}}_{j}^{r}$ means the increase of the number of sensors involved in the authentication procedure, resulting in the possibility of the adversary compromising the involving sensors and launching FEDoS attacks.

### 5.2. Performance Analysis

**Storage Overhead.** Each sensor needs to store a Bloom filter, key, and prime number. They imply the constant storage overhead. At each round *r*, each sensor *s_j_* sends ${\bar{t}}_{j}^{r}$ authentication materials, 〈*j, P, E, Z*〉, to the randomly selected sensors. As a collection interval consists of *v* rounds, we therefore know that the storage overhead of each sensor in each collection interval is $O\left(v\cdot {\bar{t}}_{j}^{r}\right)$.

**Communication and Computation Overhead.** As described in Section 5.1, the upper bound of the number of sensors involved in the authentication procedure for a single datum is $\sum_{\sigma =1}^{\lambda }\frac{\left|N_{\sigma }\right|}{n}t\sigma$. Since each involved sensor performs either sending or receiving operation, the number of involved sensors can be used to estimate the communication and computation overhead of AAD. As the size of the authentication material is constant in terms of the network size, *n*, the communication overhead of each sensor in AAD is $O\left(\sum_{\sigma =1}^{\lambda }\frac{\left|N_{\sigma }\right|}{n}t\sigma \right)$. In addition, as the number of the operations needed to be performed is also a constant in terms of the network size, the communication overhead of each sensor in AAD is $O\left(\sum_{\sigma =1}^{\lambda }\frac{\left|N_{\sigma }\right|}{n}t\sigma \right)$ as well.

## 6. Conclusions

In this paper, a scheme, called AAD, is proposed to Acquire Authentic Data in UWSNs. AAD has superior resilience against sensor compromises over the prior works. Compared with the existing methods that are vulnerable to PDoS and FEDoS attacks, AAD can also be resilient against PDoS and FEDoS attacks. Interestingly, the communication overhead, which dominates the energy consumption of sensor networks, can be even lower than that of prior works. The efficiency and effectiveness of AAD have been demonstrated *via* the analysis and simulation.

In addition to the PDoS and FEDoS attacks considered in the paper, there are actually many attacks that need to be considered too. Thus, one of future works is to equip our proposed AAD scheme with the ability to defend against radio jamming attacks. In addition, we also consider the possibility of mobile sensors in future applications. For example, the sensors may cruise a given area to collect the data of interest. For now, our method cannot apply to such scenario because our AAD scheme heavily relies on the invariant position information of each sensor. Hence, another future work is to develop a variant of AAD so that it can work on the mobile sensor networks.

## Figures and Tables

**Table 1.** Notation table.

Notation	Description
*s_j_*	The sensor *j*
*r*	The *r*-th round
$d_{j}^{r}$	The data sensed by *s_j_* at round *r*
$k_{j}^{r}$	The key used by *s_j_* at round *r*
$E_{k_{j}^{r}}\left(d_{j}^{r}\right)$	The encryption of $d_{j}^{r}$ with key $k_{j}^{r}$
$z_{j}^{r}=h_{k_{j}^{r}}\left(d_{j}^{r}\right)$	The hash value of $d_{j}^{r}$ with key $k_{j}^{r}$
$S_{j}^{r}$	A random subset of sensors selected by *s_j_* at round *r*
${\bar{t}}_{j}^{r}$	The number of co-authenticators selected by *s_j_* at round *r*
